# Against All Odds: Computational Screening Via Machine Learning Ranking and Generation of Antibody Candidates for Creutzfeldt-Jakob Disease

**DOI:** 10.17912/micropub.biology.002204

**Published:** 2026-09-04

**Authors:** Vinayan Tiruvellore, Mike Koegle

**Affiliations:** 1 College of the Canyons, Santa Clarita, California, United States; 2 Academy of the Canyons, Santa Clarita, California, United States

## Abstract

Creutzfeldt-Jakob disease (CJD) is a fatal prion disorder with no approved treatments. This study developed a machine learning pipeline trained on 21 literature-curated PrP-targeting CDR sequences to rank 29,574 original and mutation-generated candidate sequences using neutralization, selectivity, and literature-based blood-brain barrier (BBB) proxy scores. The models showed internal performance (neutralization AUC = 0.9239; selectivity AUC = 0.7759), identified 10 high-percentile candidates, and generated 7,309 novel variants. These findings support hypothesis-generating sequence-level prioritization of PrP-targeting candidates, but do not establish native PrP ^Sc^ -specific binding, full-antibody efficacy, exact PrP epitope recognition, or in vivo BBB penetration and require further experimental validation and testing.

**Figure 1. Machine Learning-Based Sequence Prioritization and Generation of Candidate Antibody CDR Sequences:** A) Machine learning pipeline for sequence-level computational prioritization of PrP-targeting CDR candidates. flow chart overview of the computational workflow used to evaluate CDR sequences against three screening criteria: blood-brain barrier compatibility proxy, selectivity-like scoring, and predicted neutralization-like activity. Background antibody sequences were scored relative to PrP-targeting exemplars, ranked by composite candidate-prioritization score, and used as the basis for mutation-based generation of novel candidate variants. B) Feature engineering and sequence-level model inputs. Quantitative physicochemical descriptors were extracted from antibody CDR sequences, including length, hydropathy, residue composition, charge distribution, aromatic fraction, and glycine/proline content. These engineered sequence features were used to train classifiers that distinguish PrP-targeting exemplar sequences from non-PrP background sequences and support multi-objective ranking. C) Model performance and candidate ranking results. Predicted neutralization-like and selectivity-like scores separated curated PrP-targeting positive exemplars from background antibodies and FDA-approved CNS antibody controls. ROC AUC and Mann-Whitney U test results support statistically significant discrimination between PrP-targeting exemplar sequences and non-PrP comparison groups. D) Structural comparison of parent and mutation-derived antibody candidates. ESMFold-predicted CDR structures show conformational effects of single-residue deletions in high-scoring mutation-derived variants. Removal of structurally restrictive residues altered loop geometry and corresponded with improved composite computational ranking relative to the parent sequences.

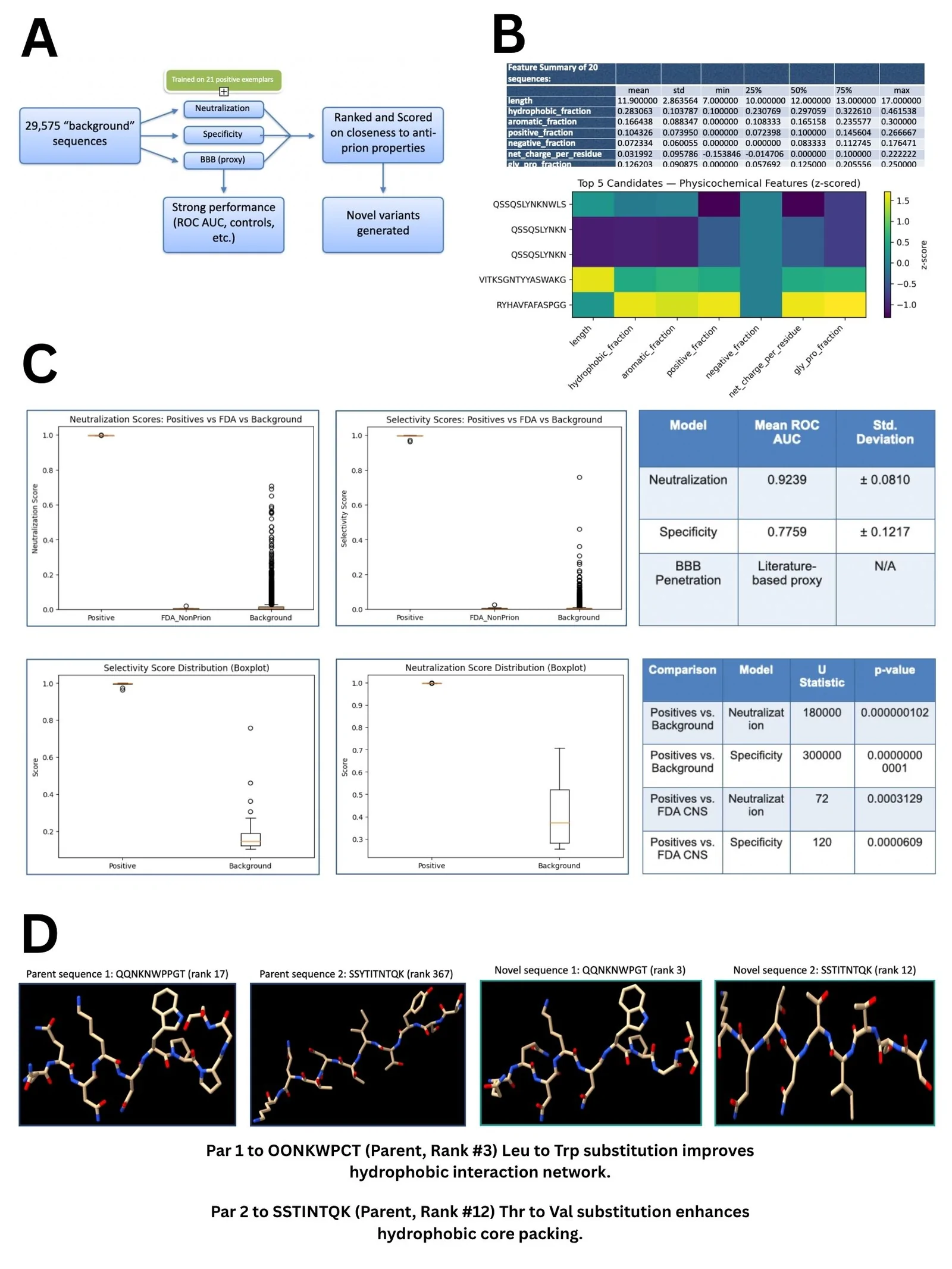



## Description

CJD is a rare but universally fatal prion disease. There is no cure, no disease-modifying therapy. CJD is caused by the misfolding of native prion protein, PrP ^C^ , into the pathogenic isoform PrP ^Sc^ . PrP ^Sc^ acts as a self-propagating template, converts normal proteins into misfolded forms, forms amyloid aggregates, and causes widespread neuronal death (Prusiner 1998; Aguzzi and Calella 2009). Therapeutic development is challenging because the pathogenic PrP ^Sc^ fold can differ substantially between species and disease-associated prion strains, so compounds effective against mouse prions may fail against human prions. Additionally, the blood-brain barrier (BBB) limits CNS delivery, and therapies must avoid disrupting normal PrP ^C^ function. Despite decades of research, no clinically approved antibody or small-molecule therapy exists (Aguzzi and Calella 2009; Pankiewicz et al. 2019).

Many reported anti-PrP antibodies act through binding to PrP ^C^ or related conformational states, and apparent PrP ^Sc^ recognition can depend on denaturation or assay-specific exposure of epitopes. Accordingly, the present study should not be interpreted as establishing selective binding to native PrP ^Sc^ . In addition, prior studies have reported neurotoxicity associated with some anti-PrP antibodies and related PrP toxic signaling pathways, emphasizing that antibody-based intervention in prion disease remains biologically complex and that the present computational rankings should not be interpreted as evidence of safety or therapeutic suitability (Sonati et al. 2013; Reimann et al. 2016; Wu et al. 2017; Frontzek et al. 2022; Mercer and Harris 2023).

Machine learning approaches to antibody engineering are promising in identifying candidate antibodies. By training ranking models on sequence-derived CDR features from curated anti-PrP antibody exemplars, it is possible to evaluate large candidate libraries and identify those most likely to satisfy computational screening criteria. The experimental question was whether a machine learning-driven computational pipeline could prioritize candidate PrP-targeting CDR segments for CJD using neutralization-like, selectivity-like, and BBB compatibility proxy criteria. The hypothesis was that a machine learning pipeline trained on sequence-derived features from literature-curated PrP-targeting CDR exemplars would rank anti-PrP-like candidates above background sequences, distinguish them from unrelated CNS antibodies, and generate novel, high-scoring variants through mutation modeling. The modeled inputs in this study were variable-length CDR segments rather than complete antibody molecules or fixed-length 10-mers. In the curated exemplar set, these CDR sequences ranged from 5 to 17 amino acids, with many clustering near approximately 10 amino acids. The framework was therefore designed for sequence-level prioritization rather than full-antibody functional prediction.

The primary control was baseline background antibodies. An external negative control used FDA-approved CNS-targeting antibodies to test whether the model captured anti-PrP sequence signatures rather than general CNS-targeting characteristics. For data collection, 21 literature-curated PrP-targeting CDR exemplars were used as positives, and more than 20,000 non-PrP sequences were used as background. Anti-PrP antibody examples and PrP-targeting antibody concepts were drawn from prior literature and patent sources (Pankiewicz et al. 2019; Uger et al. 2016; Williamson et al. 2001; Collinge and Hawke 2009). Sequences derived from Uger et al. 2016 were included as literature-reported PrP-targeting exemplars for computational comparison, but not as definitive evidence of selective native PrP ^Sc^ recognition. For feature engineering, CDR sequences were converted into quantitative descriptors, including 3-mer motif embeddings, amino acid composition, length, net charge at pH 7.4, hydropathy using the Kyte-Doolittle scale, aromatic fraction, and residue class proportions.

Model training was designed to distinguish positives from background. The pipeline included a neutralization-like classifier, a selectivity-like classifier, and a BBB compatibility proxy. Candidate ranking was performed by scoring more than 20,000 sequences and calculating a composite score across all three criteria. The top 25 candidates were selected. Mutation modeling was then applied through targeted sequence modifications, and modified sequences were re-scored to identify novel high-ranking candidates.

The neutralization-like classifier achieved ROC AUC = 0.9239 ± 0.0810, and the selectivity-like classifier achieved ROC AUC = 0.7759 ± 0.1217. Both were above random expectation, supporting that CDR sequence-derived features carry meaningful signal for PrP-targeting exemplar ranking. The BBB compatibility proxy was a literature-based heuristic informed by papers on antibody BBB transport and anti-PrP antibody fragments (Triguero et al. 1989; Yang et al. 2014; Ruiz-López et al. 2021). Strong internal model performance was achieved despite training on only 21 curated exemplars.

Mann-Whitney U tests showed statistically significant score separation between positive exemplars and background sequences and between positives and FDA CNS antibodies. Positives versus background for neutralization had a U statistic of 180000 and a p-value of 0.000000102. Positives versus background for selectivity had a U statistic of 300000 and a p-value of 0.0000000001. Positives versus FDA CNS antibodies for neutralization had a U statistic of 72 and a p-value of 0.0003129. Positives versus FDA CNS antibodies for selectivity had a U statistic of 120 and a p-value of 0.0000609. These low p-values support score separation between predefined groups, but do not independently establish antibody activity, selective native PrP ^Sc^ recognition, or guarantee model generalizability.

The neutralization score distributions showed positive exemplars clustered near the highest predicted values, around 0.99 to 1.0, whereas background sequences remained spread across lower score values, around 0.3 to 0.7. The selectivity-like score distribution showed a similar pattern, with positive exemplars again occupying the highest scoring region and background sequences occupying lower score ranges. Neutralization-like and selectivity-like scores were also compared across known PrP-targeting exemplars, FDA CNS antibodies, and background sequences. Positive exemplars clustered near maximal scores, FDA CNS antibodies scored near zero, and background sequences showed low-score distributions. Together, these comparisons support that the classifiers captured features associated with the PrP-targeting reference set rather than general antibody features.

The ranked candidate pool consisted of more than 20,000 original antibody sequences and about 7,000 mutation-generated variants. This search space allowed the machine learning models to identify rare high-scoring candidates within diverse antibody sequence space. Ten candidates ranked in the 95th to 99th percentile across 29,574 evaluated sequences. Positive exemplars were heavily enriched in the top 50, while FDA CNS antibodies were entirely absent. Mutation modeling generated 7,309 novel sequences not present in the original library. The top novel candidates ranked 3rd, 7th, and 12th overall, outperforming 99.97% of all evaluated sequences in this computational screen.

Single-residue deletions produced the largest improvements. In one case, parent sequence QQNKNWPPGT ranked 17, while novel sequence QQNKNWPGT ranked 3. A single Pro8 deletion distinguished the parent from the novel generated sequence. Removing Pro8 relieved local conformational constraint in the CDR-segment loop and improved the composite computational score from 0.9738 to 0.9810, advancing the sequence from rank 17 to rank 3 out of 29,574 total candidates. In another case, parent sequence SSYTITNTQK ranked 367, while novel sequence SSTITNTQK ranked 12. A single Tyr3 deletion distinguished the parent from the novel generated sequence and increased the combined score from 0.9439 to 0.9759, producing the largest score improvement across the highlighted mutation pairs. These examples suggest that small local sequence changes can measurably alter the ranking outputs of the computational framework.

This study shows that machine learning can prioritize and refine candidate CDR sequences for Creutzfeldt-Jakob disease despite extremely limited training data. Both predictive classifiers distinguished PrP-targeting exemplars from unrelated background sequences using only sequence-derived features. The neutralization-like model achieved strong discriminative performance, while the selectivity-like classifier demonstrated moderate yet statistically significant predictive capability. These results indicate that meaningful sequence-level signal is encoded within antibody sequence features such as residue composition, charge distribution, hydrophobicity, and short motif content.

Composite ranking across three independent computational screening constraints, including BBB compatibility proxy, anti-PrP selectivity-like scoring, and neutralization potential, allowed the pipeline to approximate candidate-prioritization trade-offs (Dobson et al. 2016; Mieczkowski et al. 2023). Rather than optimizing a single metric, the framework balances multiple biological requirements simultaneously. This multi-objective scoring approach enabled identification of candidates that perform well across all criteria rather than excelling in only one dimension.

External control comparison using FDA-approved CNS antibodies provided additional evidence that the models capture features associated with PrP-targeting rather than generic CNS-associated properties. These control antibodies scored near zero in both predictive models, while PrP-targeting exemplars clustered near the highest score ranges. This separation supports that the classifiers learned biologically relevant signal associated with PrP targeting rather than simply recognizing general CNS antibody traits.

The mutation model further expanded the search space by generating targeted sequence modifications around top-ranking candidates. Several mutation-derived variants achieved scores comparable to or exceeding their parent sequences, demonstrating the potential for computational prioritization of candidate antibody sequences. Traditional anti-PrP drug discovery is bottlenecked by the scarcity of validated antibody datasets and the difficulty of experimental prion screening. This pipeline demonstrates that sequence-level machine learning can extract meaningful biological signal from as few as 21 positive examples, but the findings remain preliminary and computational. The identified candidates represent a prioritized set for later validation, including molecular docking against PrP structures, binding affinity assays, cell-based BBB transcytosis models, and prion cell model neutralization testing. Future work would center around gathering more positive exemplars, wet-lab validation, expanding the mutation model, and replacing the BBB proxy with a trained transport model. These results should be interpreted as hypothesis for sequence ranking rather than proof of native PrP ^Sc^ -specific binding, defined PrP epitope recognition, or BBB permeation. These results should also be interpreted in light of prior reports of anti-PrP antibody-associated neurotoxicity.

## Methods

Data collection: This study was designed as a preliminary computational screening pipeline for candidate PrP-targeting CDR sequences rather than as evidence of therapeutic efficacy. The positive set consisted of 21 curated PrP-targeting CDR sequences compiled from patents and literature-derived criteria sets: 8 sequences in the neutralization set, 11 in the selectivity set, and 2 BBB-related comparator sequences. The modeled sequences represent variable-length CDR segments ranging from 5 to 17 amino acids and therefore capture only CDR-derived subregions of the antibody variable domain rather than a complete antibody molecule. Because experimentally validated anti-PrP antibody datasets are scarce, this curated set was used as a small hypothesis-generating positive reference set. For model contrast, more than 20,000 non-PrP human IGH background CDR sequences were used as negatives during training. Additionally, the source reference, sequence identity, exact sequence used, region, and model role for each exemplar are provided in exemplar_table_full.csv in the project GitHub repository.

Control construction: The primary negative control was the non-PrP IGH background set. An external comparator set consisting of 8 FDA-approved CNS-related antibodies that were not designed to bind PrP was used during downstream evaluation. These FDA CNS antibodies were treated as non-PrP comparators to test whether the scoring framework was capturing PrP-related sequence patterns rather than general CNS-targeting characteristics.

Feature engineering: Each CDR sequence was converted into a hybrid feature representation consisting of character-level 3-mer sequence features and 7 physicochemical features. The physicochemical features were length, hydrophobic fraction, aromatic fraction, positive-residue fraction, negative-residue fraction, net charge per residue, and glycine/proline fraction. In the final target feature matrix, the 21 positive sequences were represented by 137 distinct 3-mer features and 7 physicochemical features. This representation was chosen to preserve short sequence motif information while also capturing coarse biochemical properties relevant to antibody recognition and developability.

Machine-learning approach: Two binary classifiers were trained: a neutralization-like classifier and a selectivity-like classifier. Both models used L2-regularized logistic regression with class balancing and the liblinear solver. Positive labels were assigned from the curated PrP-targeting sequence set, and negative labels were assigned from the non-PrP background set. The models therefore estimated whether a sequence more closely resembled the curated neutralization or selectivity exemplars than the background repertoire. The computational framework was trained on sequence-derived features from curated PrP-targeting CDR exemplars and was not trained directly against the full human PrP ^C^ sequence. It does not predict a specific PrP binding epitope. These outputs should therefore be interpreted as ranking scores rather than direct predictions of full-antibody therapeutic activity or native PrP ^Sc^ -specific binding.

Training and validation: Model performance was evaluated using 5-fold stratified cross-validation with ROC AUC and average precision. The neutralization-like classifier achieved mean ROC AUC = 0.9239 ± 0.0810, and the selectivity-like classifier achieved mean ROC AUC = 0.7759 ± 0.1217 across folds. Because only 21 curated positive exemplars were available, these models should be interpreted as preliminary ranking models rather than definitive predictive classifiers. The larger variance observed for the selectivity model is consistent with reduced stability under limited positive-sample conditions.

BBB proxy scoring model: The BBB proxy scoring model was a hand-engineered sequence-ranking function rather than an experimentally trained BBB penetration model. For each antibody sequence, the model computed six physicochemical descriptors: sequence length, approximate molecular weight, net charge at pH 7.4, estimated isoelectric point (pI), GRAVY hydropathy (Kyte-Doolittle average hydropathy), and a hydrophobic patch score defined as the fraction of 6-residue windows containing at least 4 hydrophobic residues. The proxy then assigned a BBB compatibility score by rewarding sequences with net charge near 6.0 and pI near 9.6 using Gaussian-like soft-band terms, while penalizing excessive charge, excessively high pI, large molecular weight, high hydropathy, and high hydrophobic patchiness using smooth soft-penalty terms. The raw score was computed as:

0.35 x charge_reward + 0.25 x pI_reward - 0.15 x size_penalty - 0.15 x gravy_penalty - 0.10 x patch_penalty - 0.10 x charge_extreme_penalty - 0.10 x pI_extreme_penalty

and then transformed to a 0-1 scale with the logistic function where BBB_score = 1 / (1 + e ^(-4 * (raw - 0.3)^ )). Higher scores indicated stronger predicted BBB compatibility under this proxy, whereas lower scores indicated weaker compatibility. Because no independent BBB transcytosis or transport training dataset was used, this score should be interpreted strictly as a computational ranking proxy for sequence-level BBB compatibility rather than as direct evidence that intact antibodies would penetrate the BBB in vivo.

Statistical adequacy and overfitting: The positive reference set was small, so overfitting risk is an important limitation. To mitigate this, the classifiers used regularized logistic regression, class balancing, and 5-fold stratified cross-validation, and were evaluated against both a large background repertoire and an external FDA CNS comparator set. These steps support the use of the framework for preliminary prioritization, but they do not eliminate the need for independent experimental validation. Accordingly, all outputs should be interpreted as hypothesis-generating candidate rankings rather than validated therapeutic predictions.

Candidate ranking: Candidate sequences were ranked by combining the neutralization-like score, the selectivity-like score, and the BBB proxy score. Ranking was performed on original seed sequences and mutation-generated variants. Across 29,574 evaluated sequences in the ranking/validation workflow, the top candidates were prioritized based on combined percentile performance across the three criteria.

Statistical testing: Mann-Whitney U tests were used to compare score distributions between predefined groups, including positives versus background and positives versus FDA CNS comparators, for both neutralization-like and selectivity-like scores. These tests were used as score-separation analyses rather than proof of causal biological discrimination or native PrP ^Sc^ -specific binding.

Mutation modeling: Sequence diversification was performed computationally by targeted mutation and rescoring. This procedure generated 7,309 novel sequences not present in the original library. These mutated candidates were evaluated using the same ranking framework as the original sequences.

Data and code availability: The curated input datasets, extracted feature tables, trained scoring artifacts, ranked candidate outputs, analysis code, and the exemplar table with the CDR sequences the model was trained on are publicly available at https://github.com/Axy-lis/CJD_ML_AAO.
